# A chromosome-level reference genome of an aromatic medicinal plant *Adenosma buchneroides*

**DOI:** 10.1038/s41597-023-02571-8

**Published:** 2023-09-28

**Authors:** Hui Huang, Chen Wang, Shengji Pei, Yuhua Wang

**Affiliations:** 1 Department of Economic Plants and Biotechnology, Yunnan Key Laboratory for Wild Plant Resources, Kunming Institute of Botany, Chinese Academy of Sciences, Kunming, 650201 China; 2 Key Laboratory of Research and Utilization of Ethnomedicinal Plant Resources of Hunan Province, College of Biological and Food Engineering, Huaihua University, Huaihua, 418000 China

**Keywords:** Genome, Health care

## Abstract

*Adenosma buchneroides* Bonati, belonging to the genus *Adenosum* (Plantaginaceae), is an aromatic medicinal plant and utilized in traditional Chinese medicine. It has been widely used as plant-based repellents to prevent vector-borne diseases. However, the lack of a reference genome limits the study of conservation management and molecular biology of *A. buchneroides*. Here, we generated a chromosome-level *de novo* genome assembly of *A. buchneroides* which is a high-quality chromosome-scale assembly of aromatic medicinal plant in Plantaginaceae. The genome has a total length of 442.84 Mb with scaffold N50 of 27.98 Mb and 95.55% of the genome assigned to 14 chromosomes. BUSCO assessment yielded a completeness score of 97.2%. Furthermore, we predicted 24,367 protein-coding genes, and 95.79% of them was functionally annotated. The chromosome-scale genome of *A. buchneroides* will be a significant resource for understanding the genetic basis and evolution of active components biosynthesis, which will facilitate further study and exploit of *A. buchneroides*.

## Background & Summary

The genus *Adenosum* (Plantaginaceae) comprises 26~29 species and is native to the tropical eastern Asia and tropical Oceania, with essential oils from most of the species and traditionally used for herbal medicine. *Adenosma buchneroides* Bonati, one taxa of the genus *Adenosum*, is a well-recognized aromatic medicinal plant long favored by the Aini people in southwest of China as an insect repellent^1–3^. As mentioned in pharmacopoeia and traditional herbal medicine books, the whole plant of *A. buchneroides* has multiple pharmaceutical activities, such as anti-rheumatic, dissipate stasis, analgesia, and diminishing swelling^4^. The essential oil of *A. buchneroides* was used for the treatment of gastro-intestinal disorders, respiratory disorders and heptatitis^4,5^, and showed strong mosquito repellent activity^1^ and positive insecticidal activity against *Callosobruchus maculatus*^6^. The medicinal value of essential oil in *A. buchneroides* is attributed to its abundant active ingredients including γ-terpinene (40.26%), carvacrol (34.98%), *p*-cymene (6.60%), α-terpinene (4.05%) and carvacrol methyl ether (3.42%)^7^. There is currently a greater requirement for plant-based repellents to prevent vector-borne diseases, such as dengue, malaria, etc^8^. Up to now, many efforts on the regulation mechanism of aromatic component biosynthesis has been made in the genus *Thymus* (Lamiaceae). Though most recently, several pseudo-chromosome level genomes of Plantaginaceae were published^9–11^. The molecular basis and evolution of those components biosynthesis in *A. buchneroides* (Plantaginaceae) are rarely reported due to the lack of a high-quality reference genome.

Here, we generated a chromosome-scale assembly of *A. buchneroides*, deciphered by integrating PacBio, Illumina and Hi-C sequencing technologies. Approximately 404.03 Mb genome was assembled with the scaffold N50 length of 27.98 Mb. A total of 386.05 Mb (95.55%) of the assembled sequences were anchored to 14 pseudo-chromosomes. The genome contains 24,367 protein-coding genes, and 95.79% of them were annotated. In addition, we identified 597 miRNAs, 1,018 tRNAs, 5,202 rRNAs, and 339 snRNA. The genome assembly of *A. buchneroides* is a valuable genetic resource of aromatic medicinal plant. The results provided new insights into the molecular basis and evolution of aromatic component biosynthesis, and laid a foundation for molecular breeding and genetic conservation of *A. buchneroides*.

## Methods

**Fig. 1:**
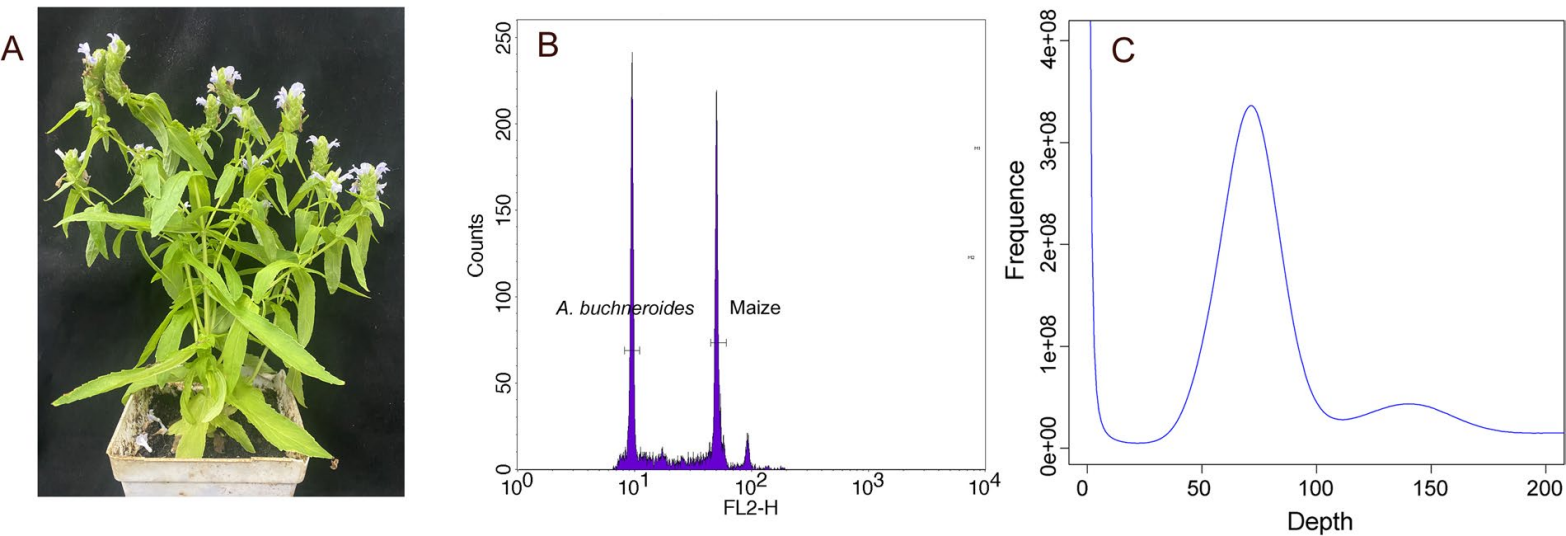
Morphology and genome size estimation of *A. buchneroides*. (**A**) Morphology of *A. buchneroides*. (**B**) Flow cytometry-based estimation. (**C**) 17-kmer distribution estimation.

Flow cytometry-based genome size estimation. The seeds of *A. buchneroides* were obtained from Mengla county of Yunnan Province, China. Seeds were germinated in a greenhouse and grown to maturity (Fig. 1A). Fresh young leaves of *A. buchneroides* were collected and immediately transferred to a pre-chilled Petri dish and chopped by a razor blade in 1.5 mL ice-cold Otto I consisting of 0.1 M citric acid, 0.5% Tween-20 with pH = 2.0-3.0^12^. The resulting suspension was thoroughly mixed and filtered through a 40 µm nylon mesh. Following incubation at room temperature for 20 min, staining solution consisting of 1 mL of Otto II solution (0.4 M Na_2_HPO_4_∙12H_2_O with pH = 8.0-9.0), 50 µg mL^−1^ propidium iodide (PI) and 50 µg mL^−1^ RNase A and 2 µL mL^−1^ β-mercaptoethanol, was added to the suspension. And then samples were kept in the dark for 30 min with occasional mixing. Flow cytometry analysis was performed in a BD FACSAria Fusion flow cytometer (BD Biosciences). Maize (2.3 Gb)^13^ was used as standard reference sample with known genome sizes. We determined that the genome size of *A. buchneroides* is approximately 439.55 ± 6.76 Mb (Fig. 1B).

Sequencing library construction and preliminary genome survey. High-quality genomic DNA was extracted from fresh young leaves of *A. buchneroides* using CTAB (cetyl trimethylammonium bromide) method. The qualified genomic DNA was broken to the target fragment (350 bp) by ultrasonic shock, and then was used to construct the short-read sequencing libraries using Illumina TruSeq^®^ Nano DNA library preparation kit (Illumina, San Diego, CA, USA). Next, paired-end sequencing was conducted on the Illumina HiSeq platform (Illumina, CA, USA), which finally generated 44.78 Gb of raw data, which covered the genome ~101.12-fold-coverage (×) (Table 1). After removing contaminants, low-quality reads and adapters by fastp software^14^, clean reads were subjected to KmerGenie^15^ for the optimal k-mer size analyzed. Then, Jellyfish^16^ was used to analyze the k-mer counts, which were used to estimate the genome size, proportion of repeat sequence and heterozygosity. From the 17-kmers distribution, we predicted that the genome size is 442.84 Mb, which is almost identical to the estimated 439.55 ± 6.76 Mb by flow cytometer. The heterozygosity and repeat ratio of *A. buchneroides* genome were predicted to be 0.28% and 58.17%, respectively (Fig. 1C). PacBio libraries were constructed using the SMRTbell template preparation kit following the manufacturer’s standard instructions, subsequently was sequenced using Single-Molecule Real Time (SMRT) sequencing on a PacBio Sequel II platform (Pacific Biosciences). In total, 40.12 Gb raw data, accounting for ~ 90.60× of the entire genome, were generated (Table 1). For Hi-C analysis, fresh leaf of *A. buchneroides* fixed with formaldehyde was used to construct library according to the protocol of Belton *et al*.^17^. The library was then sequenced on Illumina HiSeq platform, which generated 50.86 Gb raw data, accounting for ~ 114.85 × of the genome.

**Table 1:** Genome sequencing and assembly of *A. buchneroides*.

Genome-sequenceing depth (×)	
PacBio sequencing	90.60 (40.12 Gb)
Illumina sequencing	101.12 (44.78 Gb)
Hi-C	114.85 (50.86 Gb)
RNA-seq sequencing (Gb)	20.12
Estimated genome size (Mb)	442.84
Estimated heterozygosity (%)	0.28
Number of contigs	161
Total length of contigs (bp)	404,022,082
Contigs N50 (bp)	21,630,045
Longest contig (bp)	30,823,587
Contigs N90 (bp)	2,613,968
Number of scaffolds	129
Total length of scaffolds (bp)	404,025,282
Scaffolds N50 (bp)	27,977,317
Longest scaffold (bp)	37,107,577
Scaffolds N90 (bp)	21,263,299
GC content (%)	32.05
Anchored to chromosome (Mb/%)	386.05/95.55

**Fig. 2:**
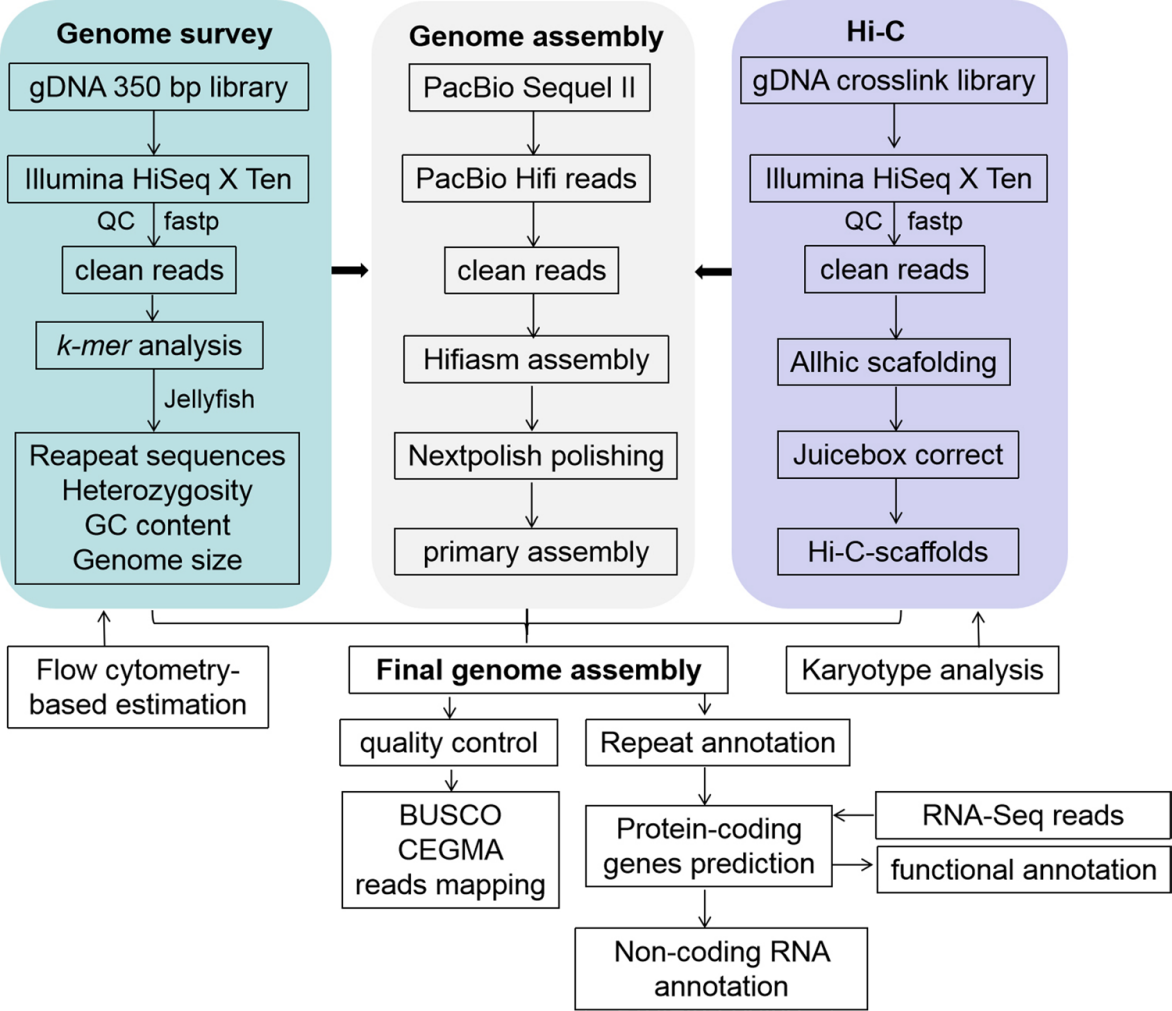
The pipelines overview of *A. buchneroides* chromosome-level genome assembly and annotation.

**Fig. 3:**
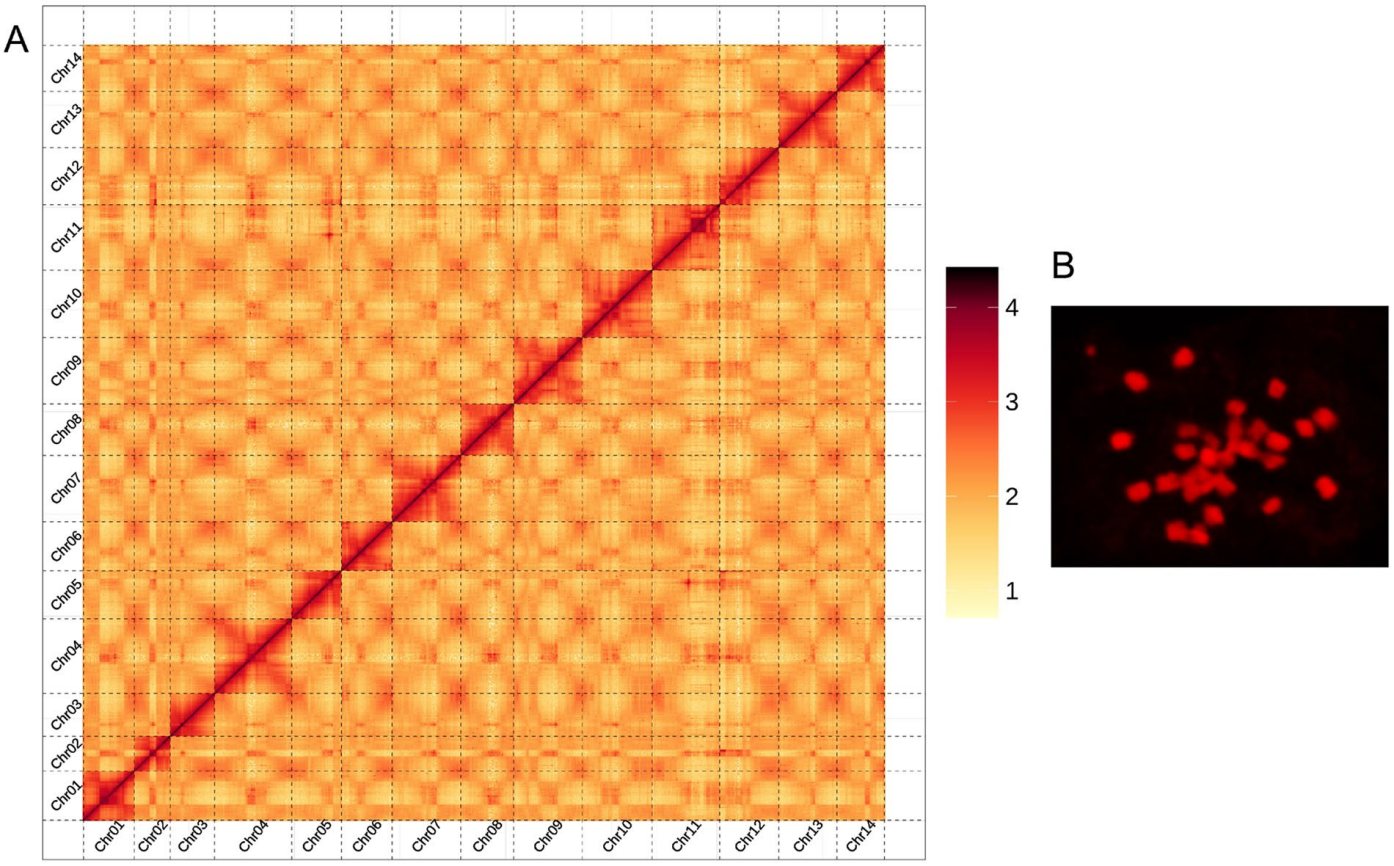
Chromosome information of *A. buchneroides*. (**A**) Hi-C interaction heatmap of *A. buchneroides* genome. Hi-C interaction matrix showing the pairwise correlations among 14 pseudomolecules. (**B**) The karyotype of *A. buchneroides*.

*De novo* genome assembly. The pipelines overview of *A. buchneroides* chromosome-level genome assembly and annotation was shown as in Fig. 2. PacBio long reads were *de novo* assembled using HiCanu v.2.2^18^ and Hifiasm v.0.13^19^, followed with polishing using NextPolish^20^. After removing low quality reads and contaminants, the high-quality Hi-C reads were used to cluster, order and orient the scaffold onto pseudo-chromosomes using the ALLHiC software v.0.9.12^21^. The Juicebox v. 201008^22^ was used to manually adjust the chromosome segmentation boundary and any wrong assembly. We preliminary assembled the PacBio long reads into 161 contigs of 404.02 Mb with N50 of 21.63 Mb, and the longest contig was 30.82 Mb (Table 1). Using Hi-C technology, these contigs were further anchored onto 14 pseudo-chromosomes, accounting for 95.55% of the assembled genome (Fig. 3A). The somatic cells of *A. buchneroides* contained 28 chromosomes by the cytological observation method (Fig. 3B). Finally, the chromosome-scale genome assembly of *A. buchneroides* was 404.03 Mb with a scaffold N50 of 27.98 Mb (Table 1).

RNA sequencing. Root, stem, leaf and flower tissue of the *A. buchneroides* were collected for RNA extraction. Total RNA was extracted from each tissue respectively using a standard Trizol protocol (Invitrogen, USA), and then used for libraries construction. After libraries construction followed the manufacture’s guideline, the transcriptomes were sequenced on Illumina HiSeq X Ten platform. In total, 20.12 Gb RNA-seq data were generated (Table 1). These RNA-seq data were used for whole-genome protein-coding gene prediction.

Repeat annotation. A combination of *ab initio* and homology-based approaches to identify the repetitive sequences. We first used LTR_FINDER v.1.05^23^, RepeatScout v.1.05^24^ and RepeatModeler v.2.0.1^25^ to build a *de novo* repeat sequences library of *A. buchneroides* genome. Then, RepeatMasker v.4.1.0^26^ was used to search for known and novel repetitive elements by mapping sequences against the *de novo* repeat library and the Repbase v.19.06^27^ database. Finally, a total of 236.86 Mb of *A. buchneroides* genome was identified as repetitive sequences, which accounted for 58.62% of the assembled genome. Specifically, four classes of transposable elements (TEs) including long terminal repeats (LTRs), long interspersed nuclear elements (LINEs), DNA elements (DNAs) and short interspersed nuclear elements (SINEs) were identified. Most of these TEs were LTRs, accounted for 49.81% of the *A. buchneroides* genome, followed by DNAs (4.75%), LINEs (0.34%) and SINEs (0.001%) (Table 2).

**Table 2:** Transposable elements (TEs) in *A. buchneroides* genome.

	Denovo + Repbase	% in Genome	TE Proteins	% in Genome	Combined TEs	% in Genome
	Length (bp)		Length (bp)		Length (bp)	
DNAs	19,194,640	4.75	12,819	0.00	19,207,066	4.75
LINEs	1,351,390	0.33	18,923	0.00	1,359,811	0.34
SINEs	283	0.00	0	0	283	0.00
LTRs	200,055,587	49.52	20,010,100	4.95	201,249,056	49.81
Unknown	36,146,034	8.95	0	0	36,146,034	8.95
Total	233,582,887	57.81	20,041,842	4.96	233,990,598	57.91

Protein-coding genes prediction and functional annotation. Prediction of protein-coding genes was based on *ab initio* gene predictions, homology-based predicitions and transcriptome-based predictions. The *ab initio* prediction was performed by Genscan v.3.1^28^, Augustus v.3.1^29^, GlimmerHMM v.1.2^30^, GeneID v.1.4^31^, and SNAP^32^ (v.2013-02-16). For homology-based prediction, BLAST v.2.10.1^33^ and Genewise software v.2.4.1^34^ were used to annotation the gene models in *A. buchneroides* using amino acid sequences from *Antirrhinum majus*, *Thymus quinquecostatus*, and *Arabidopsis thaliana* genome. For RNA-Seq-based prediction, RNA-Seq data were assembled against reference transcripts using Hisat v.2.0.4^35^ and Stringtie v.1.3.3^36^. Then, the no-reference transcripts were assembled *de novo* using Trinity v.2.1.1^37^. The results of gene prediction from three approaches were saved in GFF3 files, and then set the weight values for each annotation method. All the predicted gene structures were integrated into consensus set with EVidenceModeler v1.1.1^38^. Finally, 24,367 gene models were predicted after integrating results of the three aforementioned methods (Table 3).

**Table 3:** Prediction of protein-coding genes in *A. buchneroides* genome.

	Gene set	Number	Average transcript length (bp)	Average CDS length (bp)	Average exons per gene	Average exon length (bp)	Average intron length (bp)
*De novo*	Augustus	28,219	3,095.85	1,148.84	4.94	232.65	494.41
	GlimmerHMM	26,535	6,145.57	740.92	3.23	229.43	2,424.27
	SNAP	36,102	4,078.25	670.15	3.95	169.46	1,153.47
	Geneid	28,887	5,085.35	1,043.80	4.79	218.12	1,067.68
	Genscan	22,498	8,621.42	1,373.33	6.09	225.44	1,423.45
Homolog	Atha	19,700	3,103.95	1,161.73	4.82	241.26	509.07
	Amaj	21,008	3,374.24	1,223.49	5.14	238.13	519.78
	Tqui	21,212	3,351.55	1,229.34	5.09	241.29	518.27
RNAseq	PASA	28,822	2,966.48	1,081.97	4.70	230.43	509.97
	Transcripts	31,827	5,696.97	2,199.15	6.72	327.13	611.23
EVM		28,356	3,432.30	1,167.89	5.06	230.81	557.75
Pasa-update		28,301	3,329.41	1,159.83	4.94	234.59	550.09
Final set		24,367	3,669.94	1,267.29	5.38	235.37	548.03
Average gene length (bp)		3,669.94					
Average exon length (bp)		235.37					
Average exon number per gene		5.38					
Average intron length (bp)		548.03					

For protein-coding gene functional annotation, we aligned the predicted protein-coding gene sequences against public functional databases using BLAST (E-value 1E-5), including Swissprot, NR, KEGG, InterPro, GO and Pfam. As a result, 23,341 of protein-coding genes (95.79%) were annotated (Table 4).

**Table 4:** Functional annotation of the predicted protein-coding genes in *A. buchneroides* genome.

	Number	Percent (%)
Total	24,367	
Swissprot	20,063	82.03
NR	23,405	95.70
KEGG	18,976	77.59
InterPro	23,411	95.72
GO	15,389	62.92
Pfam	19,428	79.44
Annotated	23,341	95.79
Unannotated	1,026	4.21

Non-coding RNA annotation. We annotated four types of non-coding RNAs (ncRNAs) that were not translated into proteins, including transfer RNAs (tRNAs), ribosomal RNAs (rRNAs), microRNA (miRNAs) and small nuclear RNAs (snRNAs). The tRNAs with high confidence were predicted using tRNAscan-SE v.1.3.1^39^. The homology searching was used to predict rRNAs against plant rRNA database. Furthermore, miRNAs and snRNAs were annotated by aligning the assembled genome against the to Rfam^40^ database using Infernal software v.1.1.2^41^. Finally, we totally identified 597 miRNAs, 5,202 rRNAs, 1,018 tRNAs and 339 snRNAs in *A. buchneroides* genome (Table 5).

**Table 5:** Annotation of non-coding RNA genes in *A. buchneroides* genome.

	Type	Copy number	Average length (bp)	Total length (bp)	% of genome
miRNAs		597	175.59	104,826	0.03
tRNAs		1,018	75.46	76,814	0.03
rRNAs	rRNAs	5,202	386.25	2,009,250	0.50
	18 S	794	1,755.79	1,394,099	0.35
	28 S	3,061	139.22	426,150	0.11
	5.8 S	772	160.18	123,657	0.03
	5 S	575	113.64	65,344	0.02
snRNAs	snRNAs	339	116.71	39,563	0.01
	CD-box	229	102.79	23,539	0.01
	HACA-box	40	138.82	5,553	0.001
	splicing	68	147.26	10,014	0.002
	scaRNA	2	228.50	457	0.000
	Unknown	0	0	0	0

MicroRNA (miRNA), Transfer RNAs (tRNAs), ribosomal RNA (rRNA), small nuclear RNA (snRNA), and small Cajal body-specific RNA (scaRNA).

**Fig. 4:**
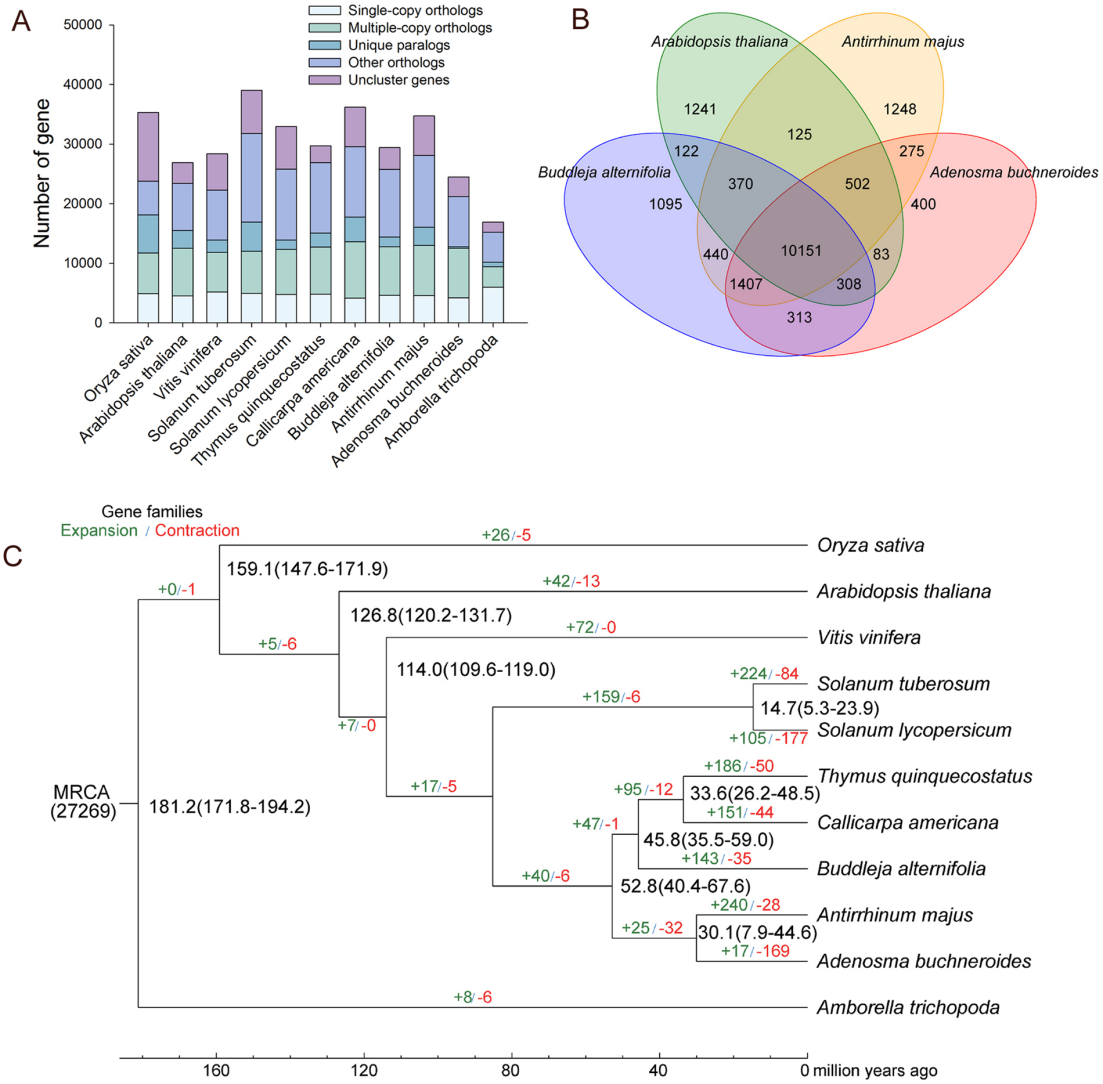
Comparative genomics and phylogenetic analyses. (**A**) Classification and statistics of common and lineage-specific genes in A*. buchneroides* and other representative plant species. (**B**) Venn diagram of orthologous genes shared among A*. buchneroides* and three other species. (**C**) Phylogenetic analysis, gene family expansion/contraction analyses and divergence time estimations. Inferred divergence times are denoted at each node in black. Gene family expansion and contraction are indicated in green and red, respectively.

Comparative genomics and phylogenetic analyses. Orthologues is critical for comparative genomics and phylogenetic analysis, and was predicted in our study. For orthologous and paralogous gene families clustering, orthologous genes of *A. buchneroides* and other 10 representative plant species, namely *A. majus*, *T. quinquecostatus*, *Callicarpa americana*, *Buddleja alternifolia*, *Solanum tuberosum*, *Solanum lycopersicum*, V*itis vinifera*, *A. thaliana*, *Oryza sativa*, and *Amborellaceae*, were analyzed through all-versus-all protein sequence similarity searches (E-value cutoff of 1E-7) using OrthoMCL software v.2.0.9^42^. We obtained the longest transcript per locus for orthologous cluster. As a result, clustering protein-coding sequences yielded 27,275 ortholog groups, including 7,145 common orthologs and 1,578 common single-copy orthologs. In *A. buchneroides*, there were 269 unique paralogs (Fig. 4A). Then, we further compared the orthologous genes among the four species including *A. buchneroides, A. majus*, *B. alternifolia* and *A. thaliana*. As shown in Fig. 4B, 10,151 ortholog genes were shared by the four species. There were 12,335 shared ortholog genes clusters between *A. buchneroides* and *A. majus*. However, there were 12,179 shared ortholog genes cluster between *A. buchneroides* and *B. alternifolia*. The result suggested that there was a closer relationship between *A. buchneroides* and *A. majus* than between *A. buchneroides* and *B. alternifolia*. Additionally, *A. buchneroides* had fewer unique gene families (400) than that in *A. majus* (1,248) in the comparison among the four species. These species-specific genes in the unique families may have close relationship with species-specific characters, and are worthy of further investigation.

We performed alignment of conserved single-copy orthologs shared by *A. buchneroides* and other 10 representative plant species with MUSCLE v3.8.31^43^. Based on these alignment, a maximum likelihood (ML) phylogenetic tree was constructed using RAxML v.8.2.12^44^. The result showed that *A. buchneroides* and *A. majus* clustered together, while the *T. quinquecostatus*, *C. americana* and *B. alternifolia* formed another cluster. These results indicated there was a closer relationship between *A. buchneroides* and *A. majus* than between *A. buchneroides* and *B. alternifolia*, in line with the result of gene family analysis. Then, we used the Bayesian related molecular clock approach in MCMCtree program with the PAML Package^45^ to estimate divergence time. The divergence times were calibrated with the TimeTree database^46^. The divergence time was as follows: *A. buchneroides-A. majus*, 30.1 million years ago (mya); *Thymus quinquecostatus*-*Callicarpa americana*, 33.6 mya; *A. buchneroides-B.alternifolia*, 52.8 mya. The divergence time between *A. buchneroides* and *A. majus* (30.1 mya) was more recent compared with the divergence time of *A. buchneroides* and *B.alternifolia* (52.8 mya). Gene families that had undergone expansion and contraction in the 11 sequenced species were determined using CAFE v3.1^47^ with a *p* value threshold = 0.05. In total, 17 and 169 gene families expanded and contracted in the *A. buchneroides* genome, respectively (Fig. 4C).

**Fig. 5:**
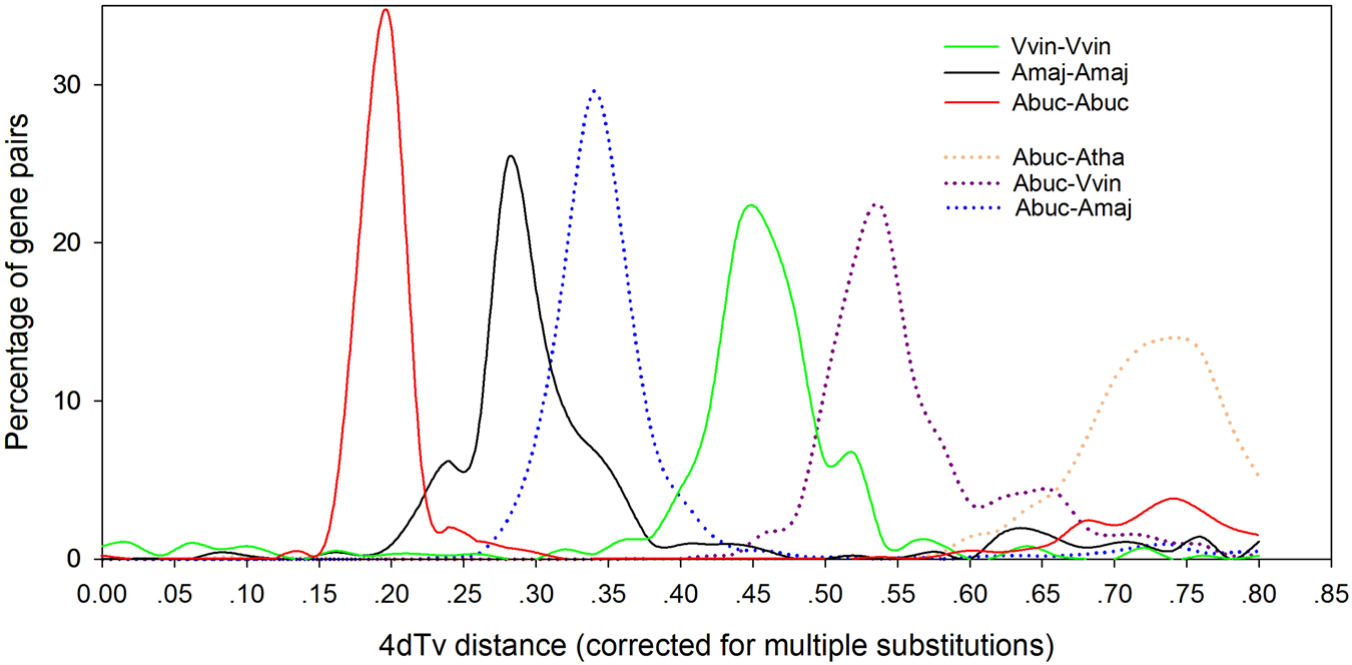
Distribution of 4DTv among *A. buchneroides* (Abuc), *A*. *majus* (Amaj), *V. vinifera* (Vvin) and *A. thaliana* (Atha) in intra- and intergenomic comparisons.

Whole-genome duplication analysis. To identify the whole-genome duplication (WGD) events in the *A. buchneroides* genome, we used MCScanX^48^ to calculated four-fold degenerated sites (4DTv) for all gene pairs. As illuminated in Fig. 5, *A. buchneroides* and *A. majus* exhibited characteristic peaks at approximately 0.20 and 0.28, respectively. The homologs of *A. buchneroides* with *A. majus* had a peak at 0.34. The results indicated a WGD event for *A. buchneroides* after divergence from *A. majus* (Fig. 5).

**Fig. 6:**
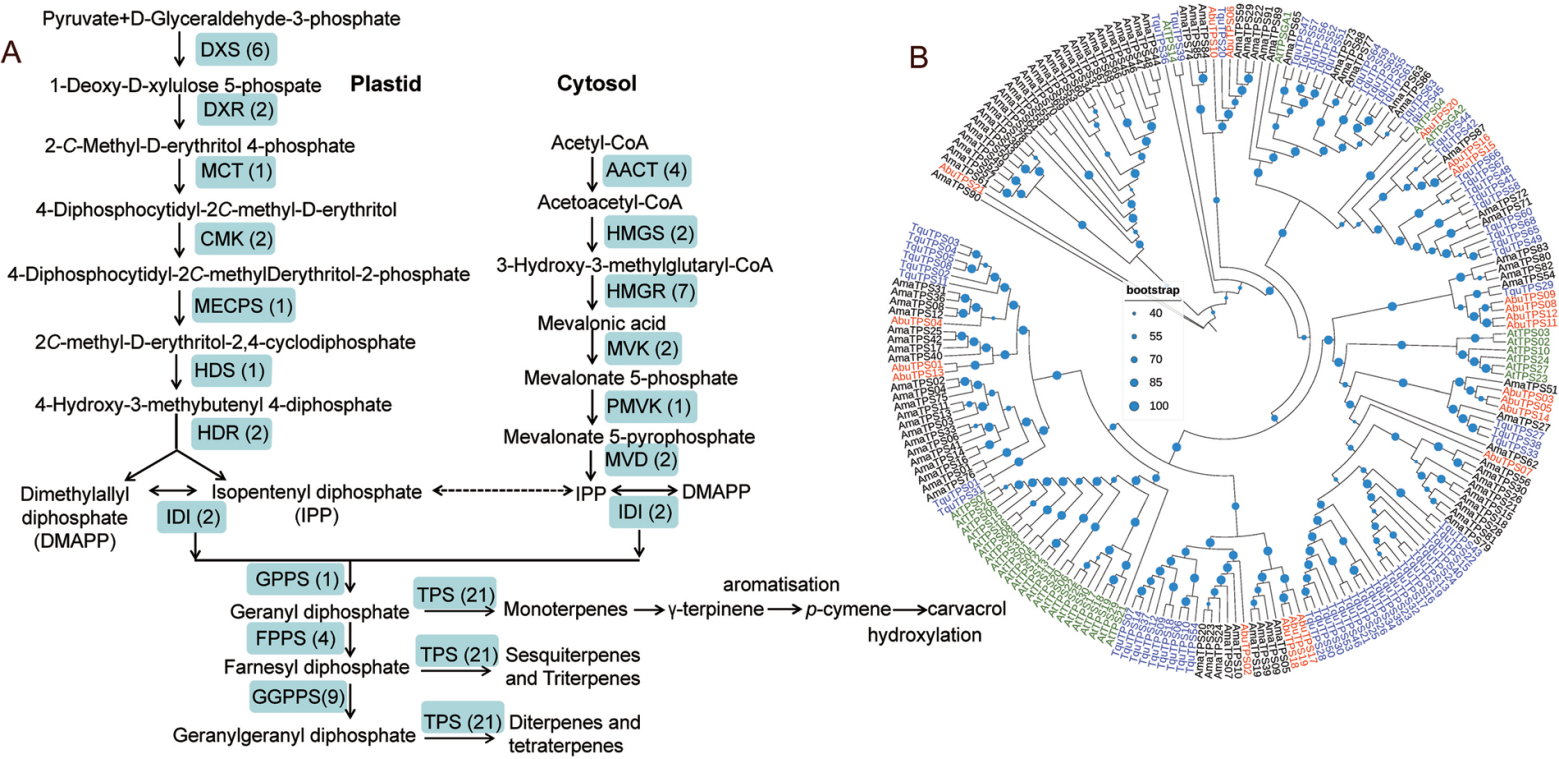
The putative biosynthetic pathway of terpenoids and gene family analysis of terpene synthases (TPSs). (**A**) The terpenodis biosynthesis pathway. (**B**) Phylogenetic analysis of TPSs from four genomes. The TPS genes of *A. buchneroides, T. quinquecostatus, A. thaliana* and *A. majus* were showed in red, blue, green and black font, respectively.

Identification of genes involved in the biosynthetic pathways of terpenoids. Previous studies reported that the medicinal value of essential oil in *A. buchneroides* was attributed to its abundant active ingredients, especially terpenoids, such as γ-terpinene and cavacrol^1,2,5^. Based on the KEGG database and the suggested biosynthesis pathways, we used a combined method of homolog searching and functional annotation to identify candidate genes for terpenoids biosynthesis (Fig. 6A). In total 70 genes in the present genome, which encoded 18 enzymes, were identified to be involved in terpenoid biosynthesis. To further explore the classification and function prediction of terpene synthases (TPSs), TPS proteins sequences in *Arabidopsis* were used as query to search against the protein database of *A. buchneroides*, *A. majus* and *T. quinquecostatus* using BLASTP program with e-value >10^−5^. All candidate proteins were further confirmed via SMART/Pfam analysis. And then all predicted TPSs were aligned with CLUSTAL. A Maximum-Likelihood (ML) phylogenetic tree was constructed by MEGA X v.10.1.7^49^, with the bootstrap values of 1000 replicates. The phylogenetic trees was imported to iTOL for visualization^50^.

## Data Records

The genome sequencing data, chromosomal assembly, genome annotations and RNA-Seq data had been deposited at the Genome Warehouse in National Genomics Data Center (NGDC), Beijing Institute of Genomics, Chinese Academy of Sciences/China National Center for Bioinformation^51^, under BioProject accession number PRJCA017315. The genome sequencing data had been deposited in the Genome Sequence Archive (GSA) of NGDC under the accession number CRA011236. The genome assembly and annotation data had been deposited in Genome Assembly Sequences and Annotations (GWH) of NGDC under accession number GWHCBPZ00000000. The genome assembly and annotations and the information of identified genes involved in terpenoid biosynthesis shown in Fig. 6 had been deposited at the figshare database^52^.

## Technical Validation

**Fig. 7:**
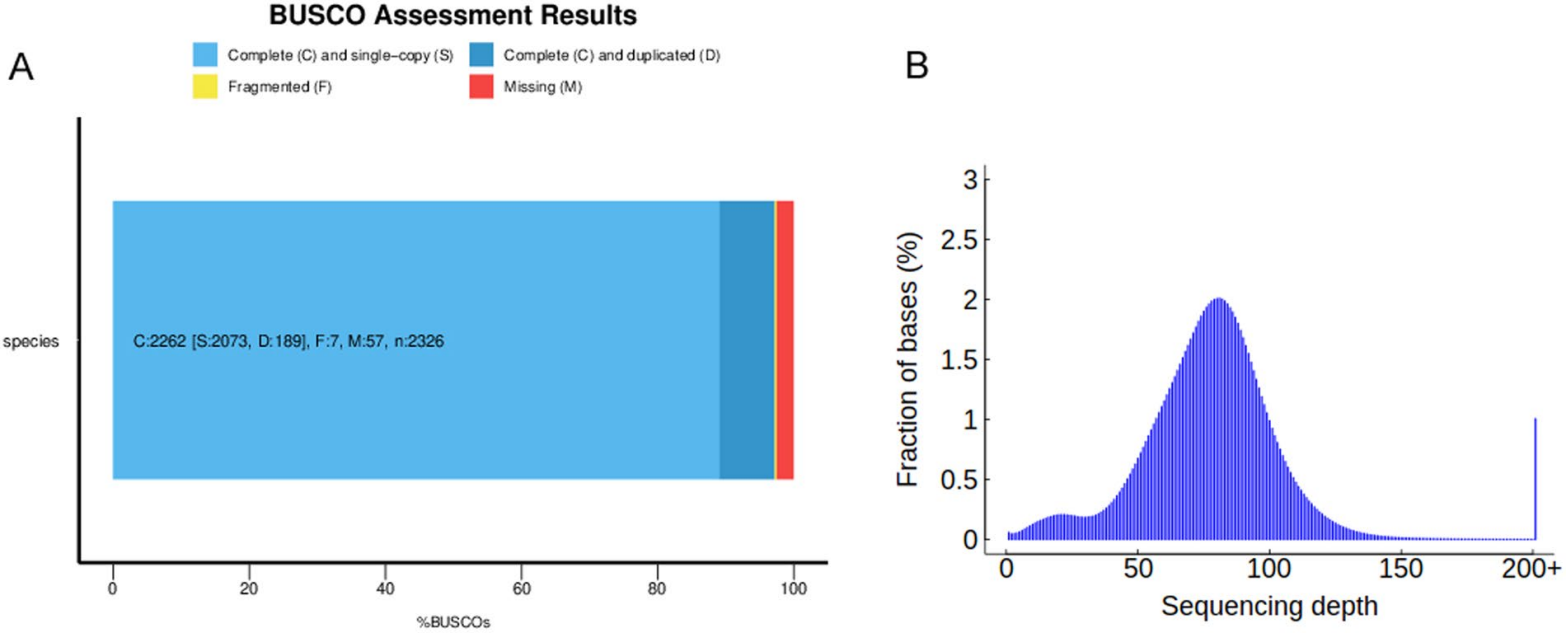
BUSCO analysis (**A**) and short Illumina reads mapping results (**B**).

The genome assembly was evaluated using Benchmarking Universal Single-Copy Orthology (BUSCO) software v.4.0.5^53^. The results revealed the retrieval of 97.25% of the complete single-copy genes, of which 8.13% were duplicated. In addition, 0.3% of BUSCO genes were fragmented, and 2.45% were missing from the genome. The BUSCO results indicated a high genome assembly completeness of *A. buchneroides* (Table 6, Fig. 7).

**Table 6:** Genome assessment of *A. buchneroides*.

	Number	Percent (%)
Completeness BUSCOs	2,262	97.2
Complete single-copy BUSCOs	2,073	89.1
Complete duplicated BUSCOs	189	8.1
CEGMA assessment	238	95.97
Reads	Mapping rate (%)	99.36
Genome	Average sequencing depth	86.47 ×
	Coverage (%)	99.84
	Coverage at least 20× (%)	97.49

Core Eukaryotic Genes Mapping Approach (CEGMA, v.2.5)^54^ employs highly conserved core eukaryotic genes (CEGs) to assess the extent of comprehensive gene coverage. The CEGMA analysis showed that the assembled genome complete recalled 238 (95.95%) of the 248 highly conserved CEGs (Table 6).

The filtered short Illumina reads were aligned back to evaluate assembly integrity and sequencing uniformity using Burrows-Wheeler Aligner (BWA) software^55^. Approximately 99.36% of the short reads mapped to the genome, and genome coverage is approximately 99.84%. By using SAMtools software^56^, we found that the ratios of heterozygous and homozygous single nucleotide polymorphisms (SNPs) were 0.001% and 1.7e-05%, respectively, indicating that the assembly had high single-base-level accuracy (Table 6).

## Data Availability

All pipeline and software used in this study were performed to data analysis according to the manuals and protocols. The parameters and the version of the software are described in the Methods section. If no detailed parameters are mentioned for a software, the default parameters were used.
